# Fatal Case of Cerebral Affection, in Which Irritability of the Stomach Was a Prominent Symptom, and in Which Tubercles Were Found in the Brain, and Softening in the Cerebellum

**Published:** 1826-07

**Authors:** 

**Affiliations:** St. George's Hospital.


*Fatal Case of Cerebral Affection, in which Irritability of the Stomach was a prominent Symptom, and in which Tubercles were found in the Brain, and Softening in the Cerebellum.* Treated by Dr. Chambers, at St. George's Hospital.

Margaret Miles, æt. forty; married; living in Suffolk-street; admitted into St. George's Hospital, March 22d, 1826.

Complains of severe shooting pains in the occiput, with slight puffiness of the pericranium, towards the left side; also of sickness of stomach, caused by taking any kind of food or drink, and by being placed in an erect posture; which position likewise aggravates the pain.

No tenderness or swelling to be felt about the epigastric region, or in any part of the abdomen. Pulse ninety, soft; tongue red, smooth, and rather dry; skin cool; bowels obstinately costive; urine free; catamenia absent for three months.

She has suffered from the headache three months: it was at first better towards the evening, but has been of late constant and severe.

℞. Hydr. subm. gr. vj.; Sacch. albi, gr. iij.
M. fiat pulvis statim et post horas duas sumendus.

℞. Aq. Menth. Sativa, ℥iss.; Magnes. sulph. ʒiss.; Tinct. Hyoscyami, m. x. M. fiat haust. quartâ quâque horâ sumend. —Beef tea.

24th.—Head somewhat relieved. Still vomits every thing she takes. Pulse eighty, soft; tongue clean and moist. Bowels opened once; motion copious, fluid, feculent, and offensive.

Hirud. viij. occipitis parti dolenti.

℞. Calomel, gr. v.; Sacch. albi, gr. iij. ter die.

25th.—Much the same.

Liq. Sodæ carbon. subinde; Calomel, gr. iij. 4tis horis.
Haust. effervescens c. Tincturæ Opii, gtt. iij. 4tis horis.

April 21st.—It is needless to continue regularly the reports of this case: it will be sufficient to say, as nothing seemed to be effectual in allaying the sickness, or otherwise materially alleviating her sufferings, that, besides being placed under the full influence of mercury, she had an issue inserted over the part of the head to which she referred particularly as the seat of pain; that she had a blister applied to the epigastrium; that she took opium, both in a liquid and a solid form, most of the anodyne extracts, the subnitrate of bismuth, &c. &c. without the slightest benefit; and at length died this day, exhausted by the progressive increase of the symptoms first mentioned.

*Sectio cadaveris*, (next day.)—Corpse much emaciated: its smell was peculiar, inasmuch as every part of it had distinctly the odour of feculency.

In the abdomen, no diseased appearance, except slight enlargement of a few of the mesenteric glands.

In the thorax, old adhesions of the right pleura: no other morbid change.

The head exhibited the following appearances:—On the calvarium and dura mater being removed, the convolutions of the cerebrum seemed somewhat flattened, as if by pressure made from within outwards. In the centre of the posterior lobe of the right hemisphere was a small soft tumor, of the size of a hazel-nut, of an ash colour, and softer than the brain. A similar mass was found in the inferior part of the posterior lobe of the left hemisphere. The substance of the brain was not hardened round these tumors. The left lobe of the cerebellum was almost entirely destroyed by the suppurative softening of a similar tumor occupying its interior. The substance of the cerebellum surrounding this abscess was indurated. There were about three ounces of serum in the ventricles, but no increase of vascularity in any part of the brain or cerebellum.

